# Promising protective potential of MiR-103a-3p against polystyrene microplastic neurotoxicity in rats

**DOI:** 10.3389/ftox.2025.1560980

**Published:** 2025-04-01

**Authors:** Leila Mohammadi, Tourandokht Baluchnejadmojarad, Mina Goudarzi, Vahid Khodashenas, Roya Khoshravesh, Mehrdad Roghani

**Affiliations:** ^1^ Department of Physiology, School of Medicine, Iran University of Medical Sciences, Tehran, Iran; ^2^ Neurophysiology Research Center, Shahed University, Tehran, Iran

**Keywords:** polystyrene microplastic, MiR-103a-3p, cognitive deficits, neurotoxicity, endoplasmic reticulum stress

## Abstract

**Introduction:** Microplastics are ubiquitous environmental pollutants with potential neurotoxic effects that can impair learning and memory. MicroRNAs are essential regulators of a number of physiological and pathological processes, but detailed information on the impact of miRNAs on the neurotoxic effects of microplastics is lacking.

**Methods:** In the present study, polystyrene microplastics (PS-MPs) were administered orally and miR-103a-3p was injected intracerebroventricularly as a treatment for PS-MPs-induced neurotoxicity.

**Results and Discussion:** Performance in the novel object discrimination Y-maze and Barnes maze tests indicated that miR-103a-3p mitigates the deleterious effects of PS-MPs on learning and memory. Oxidative stress, pyroptosis, apoptosis and inflammation induced by PS-MPs were modulated after miR- 103a-3p injection by reducing malondialdehyde, protein carbonyl, nitrite, caspase 3, caspase 1, TNFα, and NLRP3 levels in hippocampal tissue. Our results also showed that miR-103a-3p can reverse the impact of PS-MPs on astrocytic reaction and SIRT1 and BDNF levels. MiR-103a-3p alleviated PS-MPs-induced endoplasmic reticulum (ER) stress through reducing the levels of PERK, CHOP and GRP78. These findings imply that miR-103a-3p exerts a neuroprotective influence against cognitive deficits induced by exposure to PS-MPs. This is achieved by reducing inflammation, oxidative stress, apoptosis and endoplasmic reticulum stress.

## Graphical Abstract

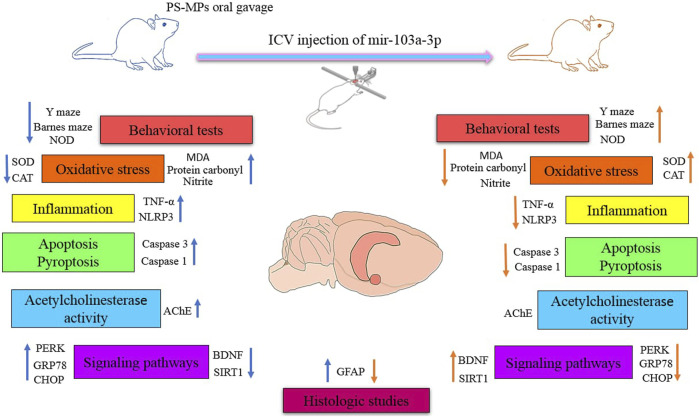

## 1 Introduction

In the realm of nature, a silent threat is lurking, Seemingly insignificant but ubiquitous: microplastics (MPs). These tiny pieces of plastic, with a diameter of smaller than 5 mm, are found in a variety of environments, including the aerosphere, marine and freshwater, soils and sediments (Sun et al., 2021; Barboza et al., 2023). They can pass through the food chain and end up in mammals, including humans (Jin et al., 2022). Recent studies have shown that polystyrene microplastics (PS-MPs) are capable of traversing the blood-brain barrier (BBB), accumulating within the brain, and inducing neurotoxicity through mechanisms involving oxidative stress and inflammation (Prüst et al., 2020; Jin et al., 2022; Gaspar et al., 2023). For example, a study in BALB/c mice showed that chronic exposure to PS-MPs led to accumulation in the brain and an inflammatory response in the hippocampus. This exposure correlated with cognitive and memory deficits as evaluated by behavioral tests such as the novel object discrimination and Morris water maze tests (Jin et al., 2022). PS-MPs also impact the cholinergic system, particularly through their effects on acetylcholine (ACh) levels and acetylcholinesterase (AChE) activity (Wang et al., 2022). In addition, PS-MPs can disrupt normal neuronal development and function by downregulating BDNF expression (Suman et al., 2024).

PS-MPs disrupt cellular homeostasis by inducing endoplasmic reticulum (ER) stress, which triggers apoptosis, inflammation and tissue damage (Wang et al., 2023b; Wang et al., 2023a). ER stress refers to the accumulation of unfolded or misfolded proteins in the endoplasmic reticulum (ER), which disrupts ER homeostasis and function (Oslowski and Urano, 2011). ER stress has been implicated as a major factor in learning and memory impairment, especially in the association with neurodegenerative disorders such as Alzheimer’s disease (AD) (Ajoolabady et al., 2022). The evidence suggests that PS-MPs induce significant ER stress through the PERK/GRP78/CHOP signaling pathway (Zhang et al., 2022). The GRP78/PERK axis has been shown to play a significant role in memory impairment associated with conditions such as Huntington’s disease (HD) (Espina et al., 2023).

MicroRNAs are short, single-stranded, non-coding RNA molecules of 21–25 nucleotides in length that are essential post-transcriptional gene regulators (Li et al., 2023). The central nervous system (CNS) contains a considerable number of miRNAs that are important for the development and function of the CNS (Rezaee et al., 2023). MiR-103a-3p is a miRNA that is involved in several processes in the nervous system, including proliferation and differentiation (Li et al., 2022). MiR-103a-3p appears to exert tissue protective effects through multiple mechanisms, including reduction of oxidative stress, apoptosis and inflammation (Zhang et al., 2019; Li et al., 2020). A study utilizing a rat model of cerebral ischemia-reperfusion (CI/R) demonstrated that miR-103a-3p exerts neuroprotective effects against brain injury (Li et al., 2020). MiR-103a-3p is also involved in the protection of cardiomyocytes under hypoxic conditions (Zhang et al., 2019). Furthermore, miR-103a-3p plays a complex role in cancer, acting as both an oncogene and a tumor suppressor, depending on the type of cancer. In hepatocellular carcinoma, for example, mir-103a-3p has been shown to promote tumorigenesis by inhibiting EVA1A (Xu et al., 2022). Conversely, studies have demonstrated that miR-103a-3p functions as a tumor suppressor in non-small cell lung cancer and prostate cancer, where it inhibits cell proliferation and invasion (Fan et al., 2019; Ge et al., 2021).

The main aim of the current study was to ascertain whether miR-103a-3p could reverse the cognitive deficits caused by exposure to PS-MPs. To investigate this, rats were orally exposed to PS-MPs for 8 weeks and miR-103a-3p was injected intracerebroventricularly (ICV) at the end of the first week. The neurobehavioral performance of the rats was then assessed at week eight using the Y-maze, novel object discrimination (NOD) and Barnes maze tests. Furthermore, we examined whether miR-103a-3p modulates the impact of PS-MPs on the induction of oxidative stress, inflammation, apoptosis and ER stress. In addition, AChE activity, GFAP immunoreactivity, and BDNF and SIRT1 expression were assessed.

## 2 Materials and methods

### 2.1 Animals

The experimental and Comparative Research Center of Iran University of Medical Sciences (Tehran, Iran) provided forty adult male Wistar rats (200–250 g). The rats were housed in groups of a maximum of four individuals per cage and kept on a 12:12 h light/dark cycle with a standard temperature (22°C) and humidity (40%–50%) and unlimited availability to food and water. The National Institutes of Health (NIH) Guidelines for the Care and Use of Laboratory Animals have been rigorously followed throughout the course of all experimental protocols and procedures. The experimental procedure also had the approval of the animal ethics committee of Iran University of Medical Sciences.

### 2.2 Experimental procedure

The rats were randomly allocated to one of the five groups: control group, miR-103a-3p control group, MP group, MP group treated with NC mimic and MP group treated with miR-103a-3p. The animals in MP group, MP group treated with NC mimic and MP group treated with miR-103a-3p were administrated 5-μm PS-MPs (Cat # 79633, polystyrene microparticles, Sigma-Aldrich, United States) via oral gavage on a daily basis for a period of 8 weeks, with a dosage of 30 mg/kg. After 1 week, the miR-103a-3p control group and the MP group treated with miR-103a-3p received miR-103a-3p (GenePharma, Shanghai, China) and the MP group treated with NC mimic received it by intracerebroventricular (ICV) injection.

**FIGURE 1:**
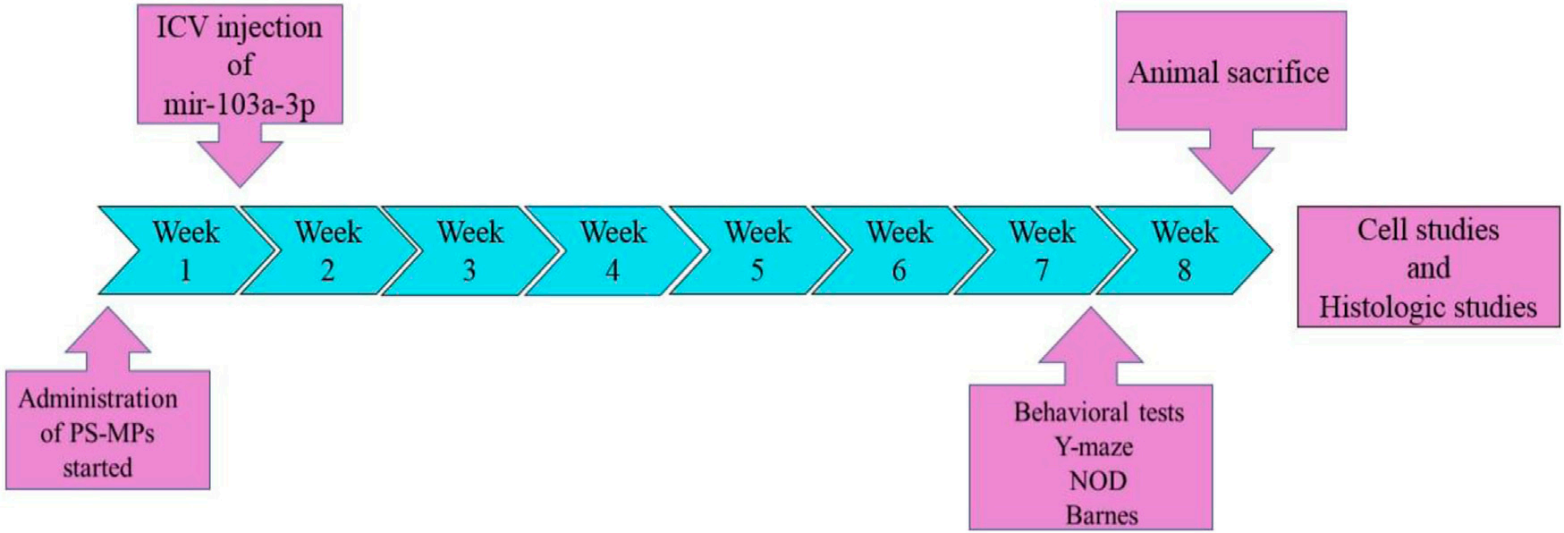
Timeline of significant events in the course of the study. Oral administration of PS-MPs from week 1 to week 8. Intraventricular injection of miR-103a-3p at the initial of week 2. Behavioral assessments (Y-maze, NOD and Barnes maze) at week 8. Animal sacrifice and eventually histological and biochemical analyses.

For this purpose, anesthesia was induced in the rats using a mixture of ketamine (100 mg/kg) and xylazine (10 mg/kg), after which they were positioned within an animal stereotaxic apparatus (Stoelting, United States). The coordinates of the injection in relation to the bregma were as follows: lateral (L) = 1.2 mm, anteroposterior (AP) = −0.2 mm, ventral (V) = 3.2 mm. In order to determine the coordinates, the Paxinos and Watson’s atlas was used as a reference. A dose of 2.5 µg/2.5 µL of mir-103a-3p was administered by intraventricular injection using a 5 µL Hamilton syringe. In the eighth week of the study, the animals’ learning and memory abilities were evaluated using three behavioral tests: Barnes maze, novel object discrimination and Y-maze. The timeline and sequence of the experiment is shown in Figure 1.

#### 2.2.1 Novel object discrimination test

The novel object discrimination (NOD) test is utilized to determine an animal’s ability to recognize new objects in its environment, which is a fundamental aspect of episodic-like memory (Antunes and Biala, 2012). To perform this test, the rats were allowed to adapt to the white Plexiglass box for a period of 24 h prior to the start of the test. After placing two analogous objects in the box, the animals were given 5 min to explore them freely. After an interval of 24 h, one of the previously observed objects was replaced with a new object. Subsequently, the animal was permitted to explore both objects for 5 min, and the time taken to explore each object was documented. To calculate the discrimination index, the following equation was utilized: (exploration time for novel object/total exploration time for novel and familiar objects).

#### 2.2.2 Barnes maze test

The Barnes maze is a tool utilized to assess rodents’ spatial memory and learning abilities (Pitts, 2018). The apparatus is constituted by a circular platform with 20 apertures distributed around its periphery. One of these provides access to an escape box. The platform, 120 cm in diameter and 1 m high, was illuminated by a 500 W bulb (1,000 lux) positioned above it to encourage the rats to seek shelter in the escape box. Visual cues, such as colored shapes, were placed around the maze to help the animals navigate and remember the location of the escape hole. For the Barnes maze task, there are 2 days of training trials followed by a probe trial. During the 2-day training trials, the rats were given up to 2 minutes to identify and enter the escape box. On the third day, the concealed chamber was takeout and the target hole was obstructed. The number of errors and the latency to escape were then noted.

#### 2.2.3 Y-maze test

The Y-maze spontaneous alternation test is an assessment of short-term memory and spatial working memory in rodents (Kim et al., 2023). The device consists of three similar arms, configured in a “Y” shape. The rat was positioned at the center of the device and permitted to explore the maze for approximately 8 min, and the sequence of arm entries was recorded. It was necessary for the animal to have both hind paws fully inside the arm. The equation (spontaneous alternation/(total number of arm entries - 2)) × 100), was used to calculate the percentage of alternation. A spontaneous alternation is the movement of a rat from one arm to another without returning to the previous arm.

### 2.3 Biochemical measurements

Following the completion of behavioral tests, anesthesia was induced in the animals via administration of ketamine (140 mg/kg) and then decapitated. The brains were quickly excised and bisected on ice. One-half of the brain was cryopreserved at −80°C for subsequent biochemical analysis. The remaining half was formalin-fixed (10%) for histological examination.

For biochemical analysis, after isolating the hippocampus on ice; it was homogenized in 150 mM Tris buffer with a pH of 7.4 using a high-speed mechanical homogenizer (IKA, Germany). After a 10-min centrifugation of the homogenates at 7,000 rpm and 4°C, the supernatant was extracted and maintained at −80°C. To perform the following tests, the supernatant was subjected to a number of analytical procedures.

In order to quantify malondialdehyde (MDA) – a marker of lipid peroxidation – 100 µL of the sample was mixed with 300 µL of TBA (thiobarbituric acid). After 60 min of heating at 95°C in a thermoblock, the mixture was allowed to cool to ambient temperature for 10 min, and subsequently centrifuged at 6,000 rpm for 5 min. Utilizing a microplate reader, the absorbance was determined at 532 nm (BioTek, United States).

A spectrophotometric method according to Reznick and Packer (1994) and Zanatta et al. (2013) was used to assess protein carbonyl (an index of protein oxidation) (Reznick and Packer, 1994; Zanatta et al., 2013). A volume of 100 µL of the supernatant was combined with 200 µL of 10 mM 2,4-dinitrophenylhydrazine (DNPH) previously dissolved in HCl. For 1 h, tubes were incubated in the absence of light. After adding 300 μL of 20% TCA (trichloroacetic acid) to each tube, the mixture was centrifuged for a period of 10 min at a temperature of 4°C and at a speed of 9,000 g. The pellet was dissolved in 225 mL guanidine (6M) in HCl (2.5N) at 37°C for 5 minutes, after washing with 0.5 mL ethanol/ethyl acetate 1:1. The analysis was then performed on the samples by a microplate reader at 365 nm.

To determine the nitrite content as an additional marker of oxidative stress, the supernatant was combined with the Griess reagent in equal volumes. The Griess reagent comprises N-naphthyl ethylenediamine and sulfanilamide and orthophosphoric acid. The absorbance was then quantified at a wavelength of 540 nm.

To measure catalase activity as a defense enzyme, 20 μL of sample was reacted with 100 μL buffer. After adding 30 µL of catalase methanol, the combination was shaken thoroughly. This was followed by the adding 20 μL of catalase substrate, and then allowed to incubate at ambient temperature for 20 min. After the addition of 30 µL of catalase stop solution and 30 µL of catalase chromogen, the mixture was left at room temperature without exposure to light. At last, 10 μL of catalase periodate was added and the absorbance was quantified after 5 min at 520–560 nm (Kiazist, Hamadan, Iran).

To quantify superoxide dismutase (SOD) as another defense enzyme, 50 µL of PBS was added to the wells of the microplate, followed by 50 µL of the sample and 10 µL of the SOD reagent. The absorbance was then recorded at 455 nm (Kiazist, Hamadan, Iran).

To measure caspase-3 activity as a marker of the apoptosis the Caspase-3 Assay Kit was used (Kiazist, Hamadan, Iran). In summary, the microplate wells were filled with 50 μL of the supernatant and then 55 μL of the working solution, which comprises caspase substrate (5 μL), caspase buffer (50 μL) and DDT (5 μL). This was allowed to incubate at a temperature of 37°C during 1.5 h and then the absorbance of the plate was read at 405 nm.

A specific kit from Abcam (United States) was employed for the purpose of quantifying caspase-1 activity. Briefly, 100 μL of reaction buffer containing 10 mM DTT was added to the wells, followed by the addition of 25 mL of supernatant. The contents of the wells were then maintained at a cold temperature. Subsequently, 10 µL of 2 mM YVAD-p-NA substrate was added and the combination was allowed to incubate for 1 hour at a temperature of 37°C without exposure to light. The absorbance was then quantified at 405 nm.

To perform acetylcholinesterase (AChE) assay, 50 μL of the supernatant was added into every well of the microplate, along with an equal volume of the standards. Following the adding of 50 μL of the acetylthiocholine reaction, the combination was incubated at ambient temperature without exposure to light for a period of 10–30 min. Subsequently, the absorbance was measured at 412 nm.

### 2.4 ELISA (enzyme-linked immunosorbent assay)

IL-10, TNF-α, SIRT1, NLRP3, PERK, GRP78 and CHOP levels in hippocampal tissue were measured utilizing ELISA. The supernatant was added to the coated microplates and incubated at a temperature of 4°C for 24 h. Following three PBS washes, the secondary HRP-conjugated antibody was added to the plates and incubated for a period of 2 h. A tetramethylbenzidine solution containing hydrogen peroxide was added to the plates after they had been washed with PBS, and the plates were incubated for a period of 15 min at ambient temperature without exposure to light. By adding 0.18 M sulfuric acid, the reaction was terminated, and a microplate reader was used to measure the samples’ absorbance at 450 nm.

### 2.5 Histological evaluation

The hippocampal blocks were embedded in paraffin, processed, and 5 μm thick hippocampal sections were prepared for subsequent experimentation.

#### 2.5.1 Nissl staining

The rat brain sections were subjected to a process of deparaffinization, rehydration and staining with 0.1% cresyl violet (Sigma Aldrich, United States). Following rinsing, dehydration, xylene transparency and Entellan coverslipping, the slides were examined under a light microscope (OLYMPUS CX41). An image-capturing and analysis system (Version 1.53, National Institutes of Health, United States) was employed to photograph Nissl-stained slides, with the density of pyramidal neurons within CA1 subsequently calculated.

#### 2.5.2 Glial fibrillary acidic protein (GFAP) immunohistochemistry

The slides were subjected to a process of deparaffinization, rehydration and washing in PBS. Following a 15-min permeation period with Triton X-100/PBS, non-specific staining was blocked by incubation with 10% normal goat serum in PBS for 1 hour at ambient temperature. Subsequently, the sections were subjected to an incubation period of 24 h with rabbit polyclonal anti-GFAP primary antibody (dilution 1/80) (SantaCruz Biotechnology, United States), under conditions of moisture and at an ambient temperature. The slides were then rinsed in PBS and incubated for a period of 2 hours with goat anti-rabbit antibody conjugated with HRP (dilution 1/140) (SantaCruz Biotechnology, United States) in PBS for 2 h. After a series of rinses, the slides were incubated for 10 min without exposure to light with 3,3-diaminobenzidine (Sigma Aldrich, United States) and 0.01% (v/v) H_2_O_2_ in PBS to allow visualization of GFAP-immunoreactive astrocytes. Subsequently, the slides were rinsed, moderately counterstained utilizing 0.1% cresyl violet, and dehydrated through a series of graded alcohols. After this procedure, the slides were cleared with xylene and a coverslip was applied with Entellan. Microscopic analysis of the slides was then performed. The assessment of astrogliosis in the hippocampal stratum radiatum was conducted on at least four sections at levels between −3.6 and −4.3 mm from bregma. To assess the reactivity of the astrocytes, integral optical density (IOD) was used.

### 2.6 Statistical methods

The data were analyzed utilizing GraphPad Prism 9 software, with one-way analysis of variance (ANOVA) followed by Tukey’s *post hoc* test. Normal distribution was ascertained through the utilization of the Shapiro-Wilk test and outliers were removed from the data sets by means of Grubbs statistical analysis. The results were reported as mean ± SEM and statistical significance was defined as a p-value of less than 0.05.

## 3 Results

### 3.1 Behavioral assessments

**FIGURE 2:**
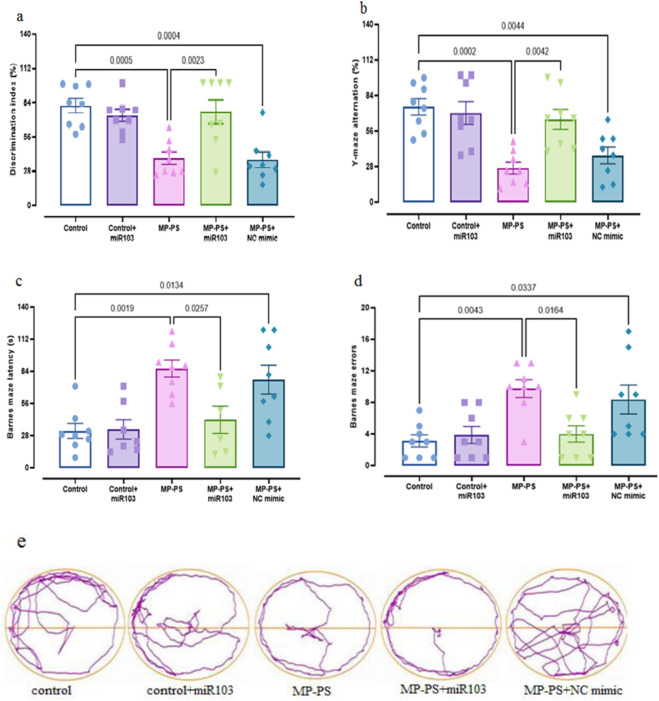
The findings of the behavioral assessments. **(a)** NOD, **(b)** Y-maze, **(c, d)** Barnes maze, **(e)** trajectories of Barnes maze. Data represent the mean ± SEM.

To determine whether PS-MPs in the rat brain affect learning and memory, Barnes maze, Y-maze and novel object discrimination tests were performed at week 8 (Figure 2).

#### 3.1.1 Novel object discrimination

A statistically significant result (F (4,35) = 8.142, p < 0.001) was observed in the NOD test (Figure 2a), with a substantial decline in the discrimination index evident in both the MP group (p < 0.001) and the MP group treated with NC mimic (p < 0.001). The decline was 52.6% and 57.8%, respectively, in relation to the control group. Conversely, in the MP group treated with miR-103a-3p, there was a remarkable increase in the discrimination index, which reached 90% compared with the MP group (p < 0.01).

#### 3.1.2 Y-maze test

Y-maze test results (Figure 2b) (F (4,35) = 9.263, p < 0.001) revealed a reduction in the alternation score of 66.5% in the MP group (p < 0.001) and 55.2% in the MP group treated with NC mimic (p < 0.01) relative to the control group. However, treatment with miR-103a-3p led to a notable elevation in the alternation score, exhibiting a 168.6% increase in comparison to the MP group (p < 0.01).

#### 3.1.3 Barnes maze test

Figure 2c, (F (4,32) = 5.722, p < 0.01) shows the escape latency and Figure 2d, (F (4,35) = 6.181, p < 0.001) shows the number of errors in the Barnes maze test. We observed a remarkable increase in escape latency of 158.9% and number of errors of 213.5% in the MP group relative to the control group (p < 0.01 and p < 0.01 respectively). Likewise, the MP group treated with NC mimic showed a 135.3% increase in escape latency and a 169.1% increase in number of errors relative to the control group (both p < 0.05). In contrast, following treatment with miR-103a-3p, a 55.9% reduction in escape latency and a 59% reduction in the number of errors was observed relative to the MP group (both p < 0.05). Figure 2e shows trajectories for the Barnes maze.

### 3.2 Hippocampal biomarkers of oxidative stress

**FIGURE 3:**
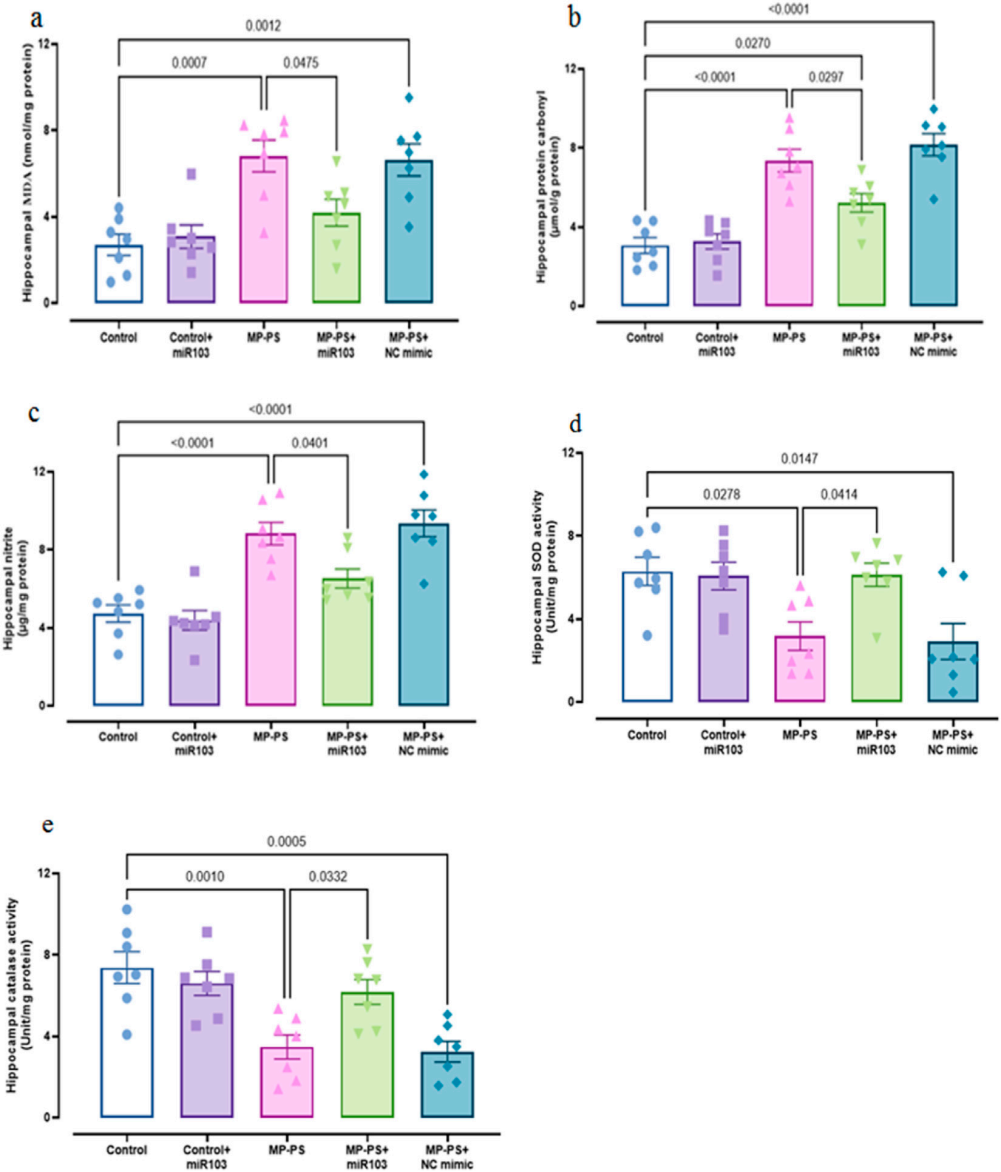
Biomarkers of oxidative stress. **(a)** MDA, **(b)** protein carbonyl, **(c)** nitrite, **(d)** SOD and **(e)** catalase. Data represent the mean ± SEM.

In order to evaluate oxidative stress, the levels of MDA (F (4,30) = 9.518, p < 0.001), protein carbonyl (F (4,30) = 23.94, p < 0.001), nitrite (F (4,30) = 19.43, p < 0.001) and activities of SOD (F (4,30) = 5.648, p < 0.01) and catalase (F (4,30) = 9.071, p < 0.001) were determined in hippocampus, which showed remarkable differences (Figure 3). In the MP group we observed an increase in MDA (p < 0.001) of 151.3%, protein carbonyl (p < 0.001) of 138.2% and nitrite (p < 0.001) of 87.1% and a decrease in SOD (p < 0.05) of 49.4% and catalase (p < 0.01) of 53% relative to the control group. Similarly, in the MP group treated with NC mimic, levels of MDA (p < 0.01) increased by 145%, protein carbonyl (p < 0.001) by 164.4% and nitrite (p < 0.001) by 98.1%, and levels of SOD (p < 0.05) decreased by 48% and catalase (p < 0.001) by 56% relative to the control group. However, ICV injection of miR-103a-3p was observed to reduce the levels of MDA (p < 0.05) by 38.5%, protein carbonyl (p < 0.05) by 28.9% and nitrite (p < 0.05) by 26.3%, and increased the levels of SOD (p < 0.05) by 86.8% and catalase (p < 0.05) by 78.4% relative to the MP group.

### 3.3 Hippocampal biomarkers of inflammation

**FIGURE 4:**
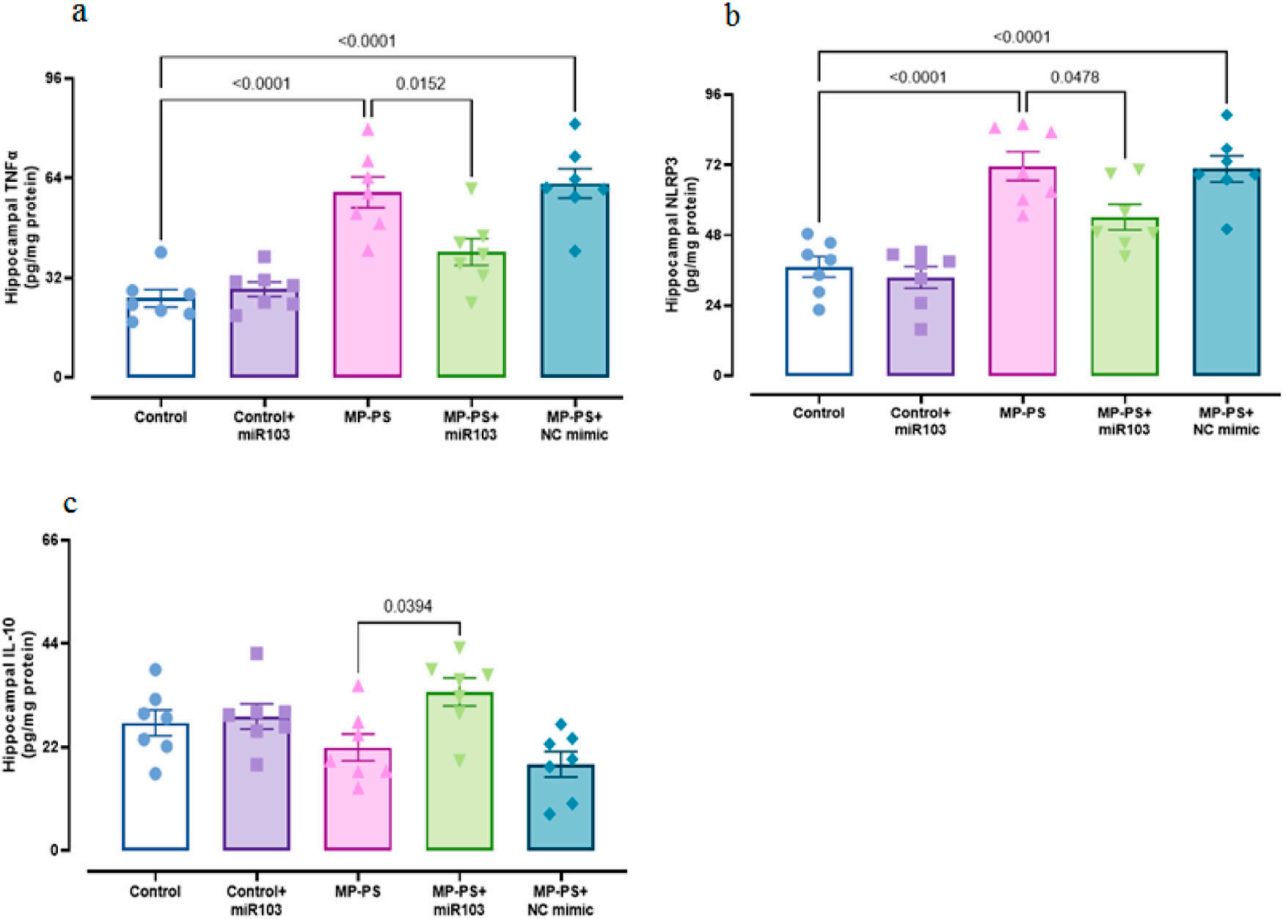
Biomarkers of inflammation. **(a)** TNFα, **(b)** NLRP3 and **(c)** IL-10. Data represent the mean ± SEM.

To assess inflammation, hippocampal levels of TNFα (F (4,30) = 18.43, p < 0.001), NLRP3 (F (4,30) = 18.85, p < 0.001) and IL-10 (F (4,30) = 4.804, p < 0.01) were measured (Figure 4). In the MP group, levels of TNFα (p < 0.001) increased by 133.8% and NLRP3 by 92.7% (p < 0.001), with no significant change in IL-10 relative to the control group. The MP group treated with NC mimic also showed an increase in TNFα of 147.9% (p < 0.001), NLRP3 of 89.1% (p < 0.001) and no significant change in IL-10 relative to the control group. However, in the MP group treated with miR-103a-3p, TNFα levels were reduced by 32.2% (p < 0.05) and NLRP3 by 24.6% (p < 0.05), and IL-10 levels were increased by 56% (p < 0.05) relative to the MP group.

### 3.4 Hippocampal biomarkers of apoptosis and pyroptosis

**FIGURE 5:**
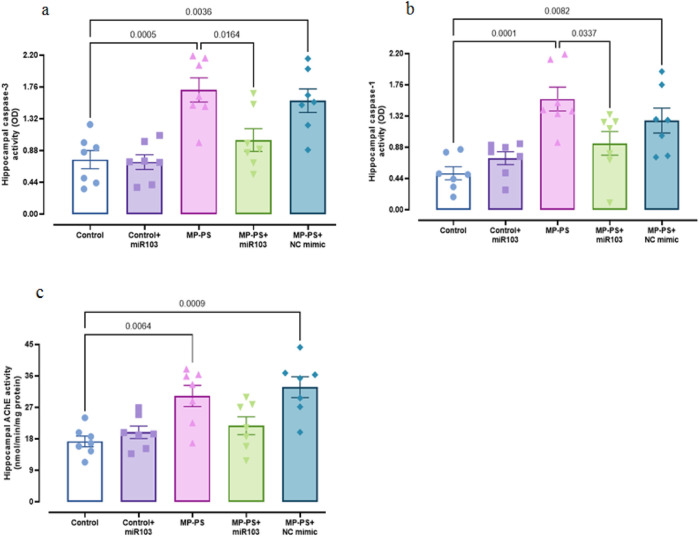
Biomarkers of apoptosis and pyroptosis, and AChE activity. **(a)** Caspase 3, **(b)** caspase 1 and **(c)** AChE activity. Data represent the mean ± SEM.

Caspase 3 (F (4,30) = 9.921, p < 0.001) and caspase 1 (F (4,30) = 8.724, p < 0.001) activity was measured as indicators of apoptosis and pyroptosis, respectively (Figures 5 a,b). The levels of caspase 3 (p < 0.001) increased by 129.3% and caspase 1 (p = 0.001) by 209.8% in the MP group relative to the control group. Likewise, in the MP group treated with NC mimic, caspase 3 was increased by 109.3% (p < 0.01) and caspase 1 by 149% (p < 0.01). In contrast, following treatment with miR-103a-3p, caspase 3 levels were observed to reduce by 40.1% (p < 0.05), and caspase 1 levels exhibited a reduction of 40.5% (p < 0.05) in comparison to the MP group.

### 3.5 Signaling pathways

**FIGURE 6:**
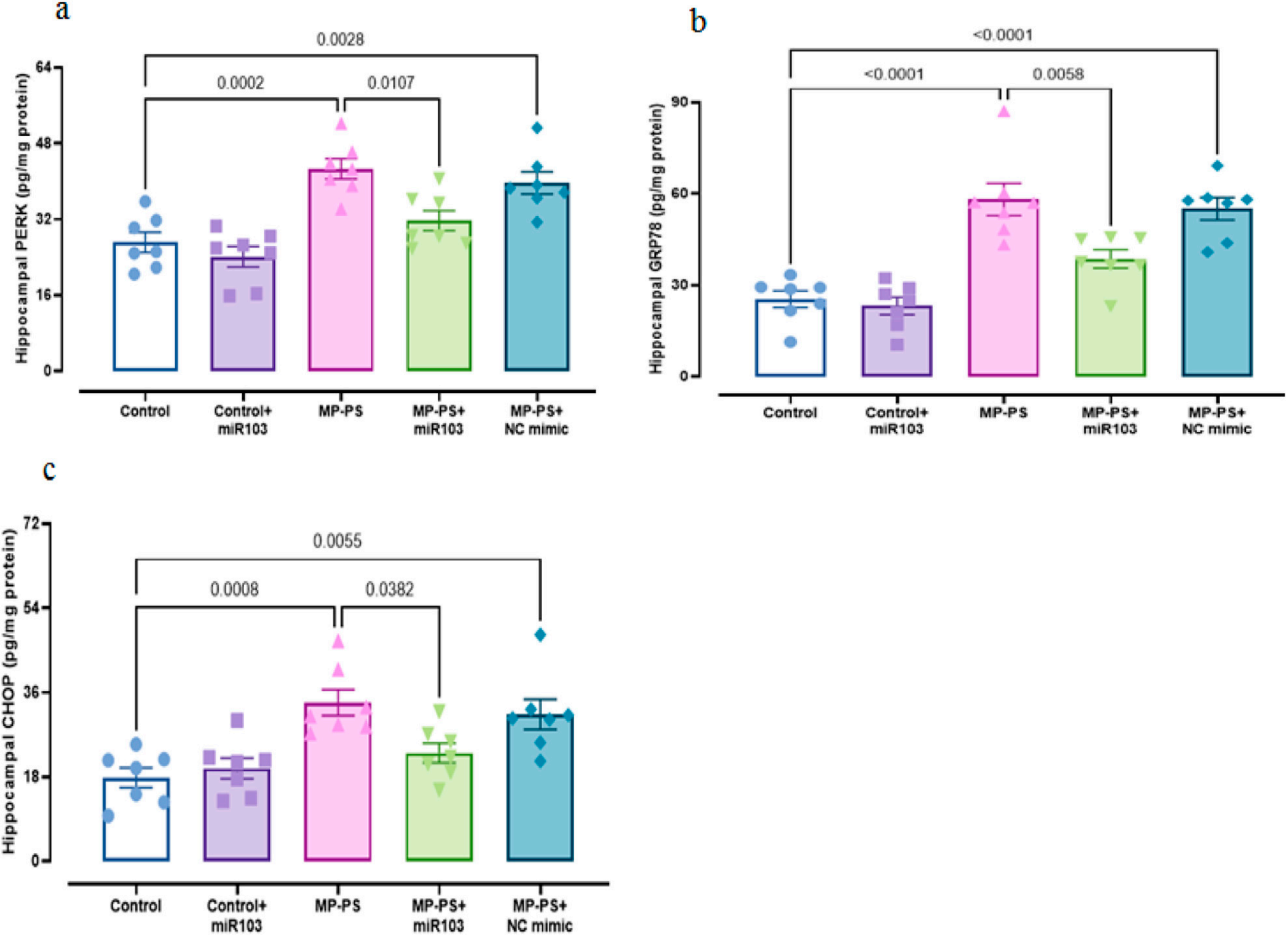
ER stress markers in hippocampal tissue. **(a)** PERK, **(b)** GRP78 and **(c)** CHOP.

**FIGURE 7:**
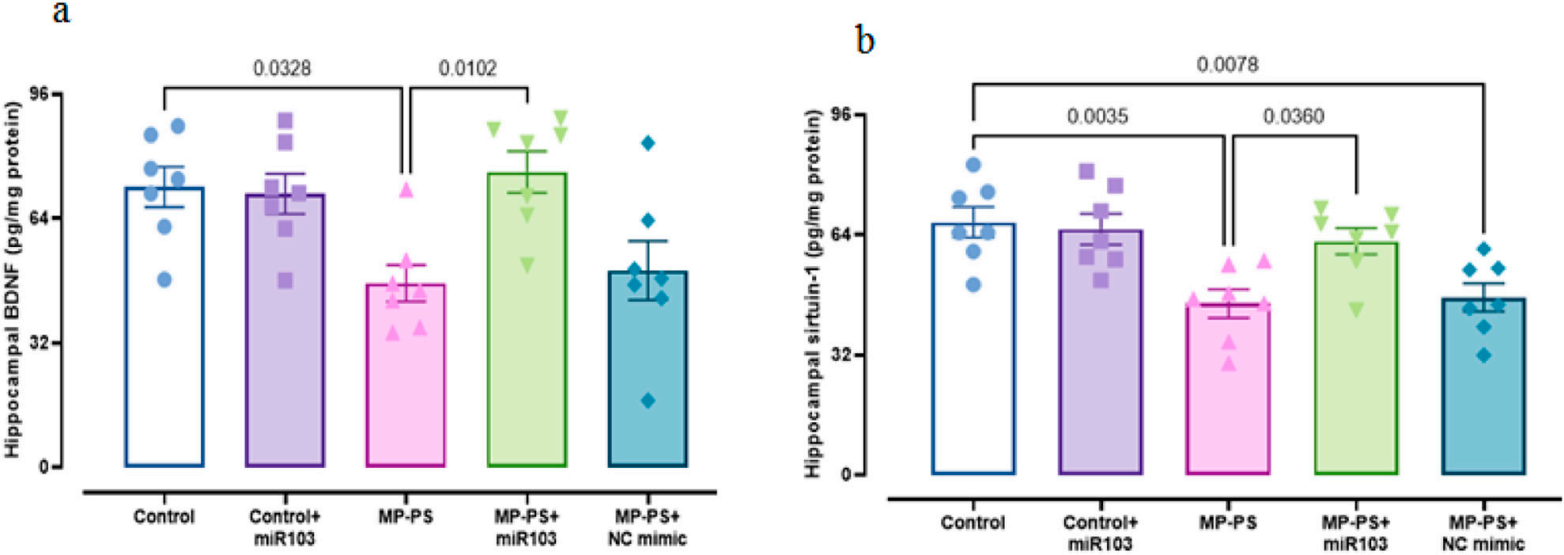
Levels of SIRT1 and BDNF in hippocampal tissue. **(a)** BDNF and **(b)** Sirtuin1. Data represent the mean ± SEM.

Signaling pathways, including PERK, GRP78, CHOP (Figure 6), Sirtuin1 and BDNF (Figure 7), exhibited remarkable differences (F (4,30) = 13.78, p < 0.001; F (4,30) = 29.91, p < 0.001; F (4,30) = 9.429, P < 0.001; F (4,30) = 7.488, P < 0.001 and F (4,30) = 5.495, P < 0.01). Compared with the control group, the MP group indicated a decline in Sirtuin1 of 32.3% (p < 0.01) and BDNF of 35.2% (p < 0.05) and an increase in CHOP of 89.1% (p < 0.001), PERK of 56.7% (p < 0.001) and GRP78 of 135% (p < 0.001). Similarly, in the MP group treated with NC mimic, Sirtuin1 (p < 0.01) decreased by 29.8% and CHOP (p < 0.01) increased by 77.1%, PERK (p < 0.01) by 46.8%, and GRP78 (p < 0.001) by 117.4%, with no significant change in BDNF. In contrast, ICV injection of miR-103a-3p resulted in a 31% increase in BDNF (p < 0.05) and a 36.8% increase in Sirtuin1 (p < 0.05), and a 31.3%, 24.9% and 35.3% decrease in CHOP (p < 0.05), PERK (p < 0.05) and GRP78 (p < 0.01) levels, respectively.

### 3.6 Hippocampal activity of AChE

The AChE activity (F (4, 30) = 8.059, p < 0.001) in the MP group was found to be notably higher by 84% than that observed in the control group (p < 0.01). Likewise, the MP group treated with the NC mimic exhibited a notable elevation in AChE activity, with an 89.8% increase relative to the control group (p < 0.001). There was no remarkable change in the MP group treated with miR-103a-3p compared to the MP group (Figure 5c).

### 3.7 Histological results

**FIGURE 8:**
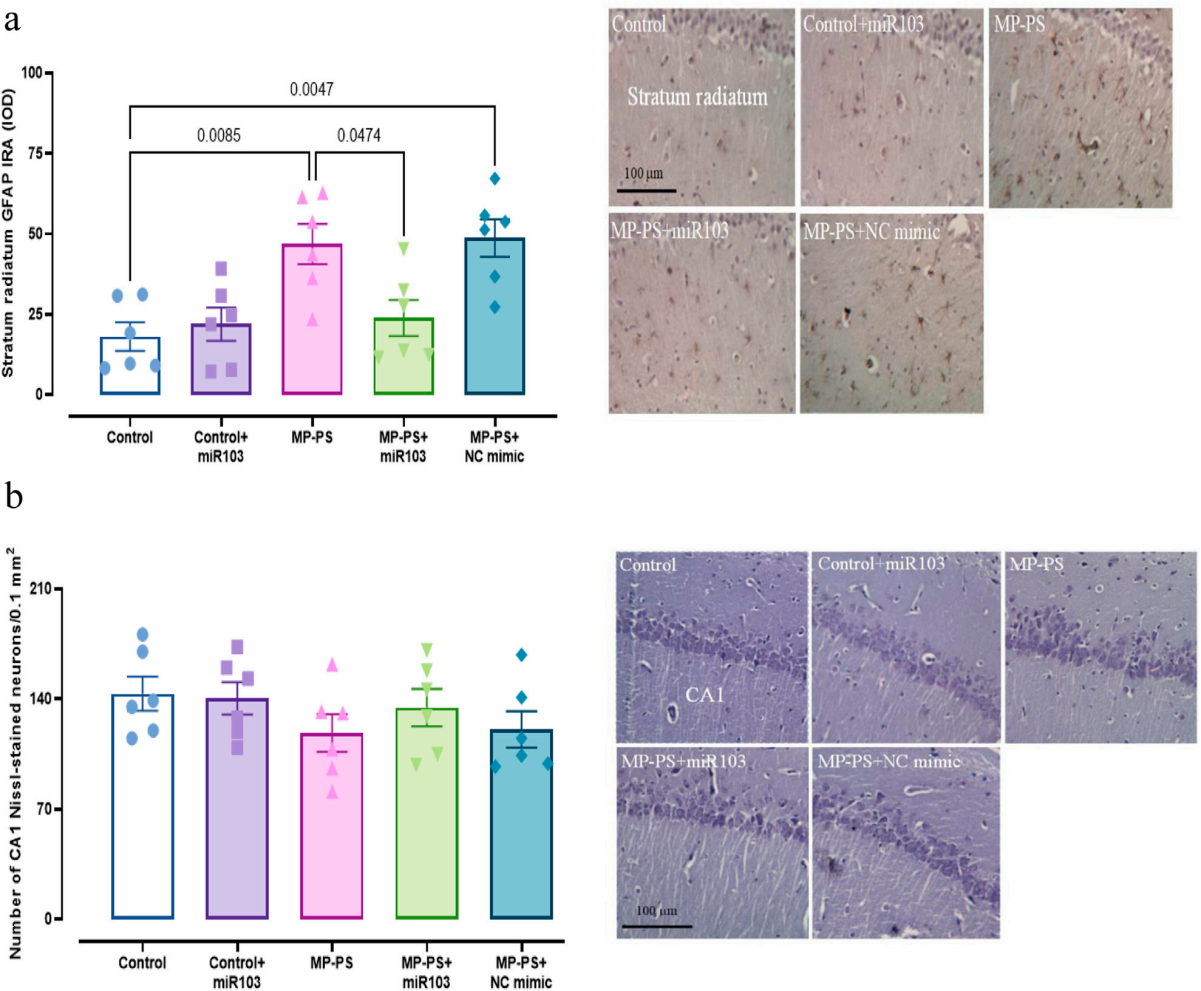
Histological analyses. **(a)** GFAP immunoreactivity in Hippocampus. **(b)** Nissl staining and statistical analysis of the number of Nissl-stained pyramidal neurons in the hippocampus. Data represent the mean ± SEM.

Figure 8a shows GFAP immunoreactivity (F (4,25) = 7.08, p < 0.01) in rat hippocampal tissue. A remarkable increase of 159.4% in immunoreactivity was observed in the MP group versus the control group (p < 0.01). Likewise, in the MP group treated with NC mimic, immunoreactivity in the MP group increased by 169.7% in relation to the control group (p < 0.01). Conversely, the injection of miR-103a-3p resulted in a 48.75% decrease in immunoreactivity compared to the MP group (p < 0.05).

The hippocampal CA1 pyramidal neuronal arrangement in the control group exhibited a normal and compact architecture. The control group that was microinjected with miR-103a-3p also exhibited a similar organizational structure. Conversely, CA1 neurons appeared irregular and less compact in rats exposed to PS-MPs. The group of PS-MPs treated with the NC mimic exhibited a similar condition. In the PS-MPs group treated with ICV miR-103a-3p, the irregular arrangement of neurons was less marked. Furthermore, no statistically significant differences were observed between the groups according to one-way ANOVA statistical analysis of CA1 pyramidal neuron density (F (4,25) = 1.01, p > 0.05) (Figure 8b).

## 4 Discussion

The results of this study confirm that ICV injection of miR-103a-3p in rats exposed to PS-MPs can reverse learning and memory deficits through modulating oxidative stress, inflammation, apoptosis, ER stress and AChE activity. There is no relevant study on the half-life of miR-103a-3p. However, the half-life of miRNAs can vary from hours to weeks. (de Rooij et al., 2022). In a study in 2023, it has been shown that a single administration of miR-145 in a model of atherosclerosis in mice can exert beneficial effects for up to 2 months. In other words, miRNAs may have long-term efficacy and sustained effects (Chin et al., 2023). Stability is an important factor in the life cycle of a miRNA. The high stability of miRNAs is necessary for their accumulation in the cell, as relatively high concentrations are required to produce their function. These molecules are relatively stable. Most miRNAs have a much longer half-life than mRNAs (Reichholf et al., 2019).

Previous studies, frequently employing the NOD test, have demonstrated that PS-MPs are linked to impairments in learning and memory processes, a finding that is in agreement with the results of our study (Lee et al., 2022; Liu et al., 2022). Furthermore, we utilized the Barnes and Y-maze tests, which serve to reinforce the validity of our results. Treatment with miR-103a-3p reversed learning and memory deficits in rats. In accordance with our findings, a prior study showed that an increase in miR-103a-3p expression was correlated with a reduction in cognitive deficits (da Silva et al., 2021). MiR-103a-3p has been shown to exert regulatory control over the expression of genes involved in neuronal development and synaptic plasticity, which are essential for the processes of learning and memory (Zheng et al., 2019; Sun et al., 2022; Rashidi et al., 2023). In consideration of the function of miR-103a-3p in astrocyte activation (Zheng et al., 2019; Rashidi et al., 2023), the mechanism by which it restores memory and learning in MP-exposed rats appears to be associated with its interaction with astrocytes. Astrocytes modulate the strength of synaptic connections between neurons, which is essential for learning and memory (Escalada et al., 2024). Dysregulation of miR-103a-3p has been observed in neurological disorders characterized by cognitive impairments, including Parkinson’s disease (PD) and Alzheimer’s disease (AD) (Zhang et al., 2020; Nies et al., 2021).

In the present study, we observed a decline in antioxidant enzyme activity, SOD and catalase, and an elevation in MDA, nitrite and protein carbonyl levels in hippocampal tissue of the PS-MPs group, which is in agreement with previous research studies (Zou et al., 2023). However, ICV injection of miR-103a-3p enhanced SOD and catalase activities and diminished MDA, nitrite and protein carbonyl levels. MiR-103a-3p has been demonstrated to exert a pivotal regulatory function in the context of oxidative stress. In a study of CI/R injury in rats, miR-103a-3p overexpression significantly increased levels of the antioxidant enzyme SOD and decreased levels of the oxidative stress marker MDA, suggesting that miR-103a-3p alleviates oxidative stress in CI/R (Li et al., 2020).

Exposure to PS-MPs also induces neuroinflammation in the hippocampus, as evidenced by the elevated levels of NLRP3 and TNF-α observed in the hippocampus of PS-MPs rats. Nevertheless, the levels of TNF-α and NLRP3 were diminished following treatment with miR-103a-3p. MiR-103a-3p is implicated in the regulation of cytokines such as TNFα. According to reports, miR-103a-3p targets these pro-inflammatory cytokines, thereby contributing to the modulation of the inflammatory response (Costantini et al., 2023).

In a previous study, it was shown that PS-MPs can induce pyroptosis and apoptosis in ovarian granulosa cells via the NLRP3/Caspase-1 signaling pathway (Hou et al., 2021). In our study, we also observed a notable increase in apoptosis and pyroptosis in the hippocampal tissue of tats in the PS-MPs group. After treatment with miR-103a-3p there was a decrease in pyroptosis and apoptosis. A previous study has shown that miR-103a-3p plays an important role in modulating apoptosis by targeting key genes involved in the apoptotic pathway (Li et al., 2020). Other studies show that miR-103 suppresses apoptosis in models of Alzheimer’s disease (AD) and spinal cord injury (SCI) (Li et al., 2018; Yang et al., 2018). In models of colitis, elevated levels of miR-103a-3p were associated with reduced inflammation and pyroptosis, suggesting that it may serve as a protective factor against inflammatory damage (Zhao et al., 2022).

Inflammation, oxidative stress, apoptosis and pyroptosis are interconnected processes that significantly impact learning and memory (Naomi et al., 2023; Yang and Tang, 2023). Chronic inflammation can lead to oxidative stress, which in turn causes cellular damage and apoptosis (Solleiro-Villavicencio and Rivas-Arancibia, 2018). Additionally, pyroptosis is a consequence of inflammatory processes (Pan et al., 2022). These processes can disrupt synaptic plasticity and neuronal function, ultimately impairing learning and memory (Naomi et al., 2023; Yang and Tang, 2023). It can be concluded that MPs have an adverse effect on learning and memory abilities, which is caused by the induction of oxidative stress, inflammation, apoptosis and pyroptosis. Conversely, miR-103a-3p restores memory and learning in rats exposed to MPs, due to its anti-inflammatory, antioxidative, anti-apoptotic and anti-pyroptosis properties.

Our results showed that oral administration of PS-MPs reduces sirt1 levels, which has been confirmed by a recent study (Gupta et al., 2023). SIRT1 enhances synaptic plasticity, thereby improving cognitive function (Liu et al., 2024). Moreover, SIRT1 activation has been shown to increase the expression BDNF, which support the growth and maintenance of neurons involved in learning and memory (Wang F. et al., 2023). PS-MPs have been shown to reduce BDNF levels (Suman et al., 2024), a finding we also observed in our research. After ICV injection of miR-103a-3p, we observed an increase in BDNF and SIRT1 in hippocampal tissue. A previous study suggested that BDNF is a target gene of miR-103a and that miR-103a may negatively regulate BDNF (Wang et al., 2018). To date, no research has been conducted on the effect of miR-103a-3p on SIRT1. However, studies of other miRNAs have shown that miRNAs are able to regulate SIRT1 expression. While the majority of miRNAs appear to suppress SIRT1 expression, some also appear to indirectly increase it (Saunders et al., 2010; Ghafouri-Fard et al., 2023; Chen et al., 2013). The established role of the SIRT1-BDNF pathway in improving learning and memory (Wang F. et al., 2023), coupled with the observed elevation in BDNF and SIRT1 levels following miR-103a-3p injection, indicates that miR-103a-3p may possess the capacity to optimize memory and learning by regulating SIRT1 and BDNF levels.

Several studies have revealed that exposure to PS-MPs results in a decrease in GFAP levels (Zhu et al., 2022; Gaspar et al., 2023). One study indicated that PS-MPs in the presence of iron elevated GFAP levels (Liu et al., 2022), whereas another study reported no change in GFAP expression (Marcellus et al., 2024). In this study, we found exposure to PS-MPs increased GFAP levels. After treatment with miR-103a-3p, we observed a decrease in GFAP levels. GFAP is a critical marker of astrocytic activity, which is associated with neuroinflammation that can affect learning and memory (Bondan et al., 2019). This suggests that PS-MPs interfere with learning and memory by increasing GFAP expression.

The findings of this study indicate that AChE activity in the hippocampus increased following exposure to PS-MPs. Previous studies have shown that microplastics can have both inhibitory and enhancing effects on AChE activity (Gambardella et al., 2017; Chen et al., 2020; Wu et al., 2022). The main reason for this contradiction seems to be the concentration of MPs. Other reasons for this discrepancy may be the size of the MPs, the duration of exposure to MPs, experimental conditions, methodological limitations and species-specific responses. After treatment with miR-103a-3p, we did not observe any change in AChE activity. There is currently no direct evidence to suggest that miR-103a-3p has an effect on AChE activity. Further research is needed to determine whether there is a correlation between miR-103a-3p and AChE activity. Despite the lack of studies demonstrating the effect of miR-103a-3p on ACHE activity, research on other miRNAs has indicated that they regulate AChE activity by targeting AChE messenger RNA (mRNA) (Mishra et al., 2017; Hanin et al., 2014). AChE plays a crucial role in modulating cholinergic transmission, which is essential for cognitive function (Chen et al., 2022). Optimal levels of AChE are necessary for efficient learning and memory formation. Inhibition of AChE activity improves memory, while excessive AChE activity impairs it (Aluko, 2021). As previously stated, the current study showed that PS-MPs elevated AChE activity. It can therefore be postulated that PS-MPs impair learning and memory by increasing AChE activity.

It has been demonstrated that exposure to PS-MPs results in the induction of ER stress, which is associated with alterations in the expression of proteins involved in the PERK/GRP78/CHOP signaling pathway (Lu et al., 2024). In our study exposure to PS-MPs increased the levels of PERK, GRP78 and CHOP. The expression of PERK, GRP78 and CHOP was reduced by treatment with miR-103a-3p. This finding is in accordance with previous research which has demonstrated that miR-103a-3p plays a role in regulating ER stress in colorectal cancer (Zhang et al., 2021). ER stress has been linked to neurodegenerative diseases, where it contributes to cognitive decline and memory impairment (Briggs et al., 2017). In a study involving HD (Huntington’s disease) mice, it was found that increased levels of GRP78 and CHOP in the hippocampus correlated with cognitive deficits (Espina et al., 2023). Taken together, PS-MPs can impair learning and memory by inducing ER stress, and miR-103a-3p can restore learning and memory by reducing ER stress.

## 5 Conclusion

The results of our investigation indicate that miR-103a-3p exerts a neuroprotective influence against cognitive impairment resulting from exposure to PS-MPs. This is accomplished by attenuating inflammatory responses, oxidative stress, apoptosis, pyroptosis and ER stress.

### 5.1 Limitations

It is acknowledged that the present study is not without limitations. One notable limitation of our study is the absence of complementary testing methods, particularly the Western blot technique. While our primary methods provided valuable insights, incorporating Western blot could have enhanced the robustness of our findings. Furthermore, we have shown that miR-103a-3p exerts a modulatory effect on the adverse effects of MPs; however, the underlying mechanism of action remains to be further elucidated.

## Data Availability

The original contributions presented in the study are included in the article/supplementary material, further inquiries can be directed to the corresponding author.

## Abbreviations

AChE, Acetylcholinesterase; BDNF, Brain-derived neurotrophic factor; CHOP, C/EBP Homologous Protein; GFAP, Glial fibrillary acidic protein; GRP78, Glucose-Regulated Protein 78; ER, Endoplasmic reticulum; ICV, Intracerebroventricularly; MDA, Malondialdehyde; abrNLRP3 Nod-like receptor protein 3; NOD, Novel object discrimination; PERK, Protein kinase RNA-like ER kinase; PS-MPs, Polystyrene microplastics; SIRT1, Sirtuin1; SOD, Superoxide dismutase; TNFα, Tumor necrosis factor-α.
